# Association Between Temporomandibular Disorders and Irritable Bowel Syndrome: A Scoping Review

**DOI:** 10.3390/jcm13237326

**Published:** 2024-12-02

**Authors:** Klara Saczuk, Sylwia Roszuk, Malgorzata Wirkijowska, Adam Fabisiak, Tan Fırat Eyüboğlu, Mutlu Özcan, Monika Lukomska-Szymanska

**Affiliations:** 1 Department of General Dentistry, Medical University of Lodz, 251 Pomorska St., 92-213 Lodz, Poland; klara.saczuk@umed.lodz.pl; 2 Division of Dentistry, Faculty of Medicine, Medical University of Lodz, 251 Pomorska St., 92-213 Lodz, Poland; sylwia.roszuk@gmail.com (S.R.); wirkijowskam@gmail.com (M.W.); 3 Department of Digestive Tract Diseases, Medical University of Lodz, 22 Kopcinskiego St., 90-153 Lodz, Poland; adam.fabisiak@umed.lodz.pl; 4 Department of Endodontics, Faculty of Dentistry, Medipol University, Cibali Mah. Ataturk Bulv. No: 27, Fatih, Istanbul 34083, Türkiye; tfeyuboglu@yahoo.com; 5 Clinic of Masticatory Disorders and Dental Biomaterials, Center for Dental Medicine, University of Zurich, Plattenstrasse 11, 8032 Zurich, Switzerland; mutluozcan@hotmail.com

**Keywords:** comorbidity, irritable bowel syndrome, stress, temporomandibular disorders

## Abstract

Temporomandibular disorders (TMDs) encompass various clinical conditions associated with the temporomandibular joint (TMJ) and the masticatory muscles. TMD symptoms include pain in the orofacial region, restricted or altered mandibular movement, and sounds associated with the temporomandibular joint (TMJ). This condition adversely affects quality of life, social functioning, and daily activities, and may also contribute to widespread pain syndromes and comorbidities, including irritable bowel syndrome (IBS). IBS is a common chronic functional disorder of the lower gastrointestinal tract, characterized by recurrent abdominal pain associated with impaired bowel symptoms. Previous studies indicate an association between TMD and IBS. This scoping review examined the correlation between TMD and IBS concerning their pathology, frequency, and severity, and the potential similarities in how the nervous and endocrine systems influence them. PubMed, SCOPUS, Web of Science, and Google Scholar search engines were utilized to identify suitable studies for this article. Following the application of selection criteria, a total of 58 clinical papers met the eligibility requirements for inclusion in the systematic review. Research showed that both conditions significantly enhance the development of one another and have mutual comorbidities. Both ailments were proven to modify central nervous system processing, leading to high comorbidity in patients. Combining dental and gastroenterological treatments, including a simultaneous therapeutic approach, can significantly enhance patients’ quality of life, but further research is needed for a holistic approach.

## 1. Introduction

### 1.1. TMD

Temporomandibular disorders (TMDs) include different clinical conditions related to the temporomandibular joint (TMJ) and masticatory muscles. The main symptoms are pain in the orofacial area, limitations or changes in mandibular movement, and TMJ sounds [1,2,3]. This condition impairs quality of life, social functioning, and daily activities, and can also promote widespread pain syndromes and comorbidities such as irritable bowel syndrome (IBS) [2,3,4]. Management of TMD-related pain depends on the patient’s clinical manifestations and includes medication, physiotherapy, manual therapy, acupuncture, electrotherapy, cognitive behavioral therapy, relaxation techniques, occlusal splints, occlusion correction, and surgical interventions [5]. The incidence of TMD ranges from 5% to 12% in the general population [3]. Only 7% to 15% of people with TMD seek medical help, and the majority of them are women of reproductive age [1].

The diagnosis of the TMD patient is complex, as individuals frequently report symptoms associated with body regions distinct from the orofacial area [2]. The Axis I is a recommended diagnostic criterion within the TMD (DC/TMD) protocol, utilized to identify pain-related TMD and to differentiate between the most prevalent pain-related TMD conditions and an intra-articular disorder [3]. The Axis II comprises screening and comprehensive self-report instrument sets. It assesses pain intensity, psychosocial distress, jaw function, parafunctional behaviors, and pain-related disability [3].

TMD rarely occurs in isolation, and 70% of patients with painful TMD suffer from at least one comorbidity [6,7,8]. Facial pain is related to abdominal pain and higher levels of depression, perceived stress levels, and further mental health issues [1,9,10]. In adolescents, TMD was found to be associated with other painful body areas and a higher number of comorbidities than in adolescents without this condition, with 11.3% of them reporting gastrointestinal disorders [9,10]. It can also be both a manifestation and a promoting factor of widespread syndromes such as fibromyalgia (FM), IBS, or whiplash-associated disorders [4,11,12,13].

### 1.2. IBS

IBS is a frequent chronic functional disorder of the lower gastrointestinal tract characterized by recurrent abdominal pain related to impaired bowel habits, and is not otherwise related to any structural or biochemical abnormalities that are possible to detect [1,14,15].

The prevalence of this condition is 11% in the global population, with women being two to three times more likely to develop IBS symptoms than men [2,16]. IBS is, therefore, seen as a significant women’s health issue [17]. Patients with this condition are typically diagnosed before the age of 45 [2]. Today, Rome IV criteria are used to diagnose IBS: abdominal pain must be present on at least 1 day/week during the last 3 months and should be related to two or more of the following criteria: (i) improvement or worsening of pain after defecation; (ii) change in stool frequency; (iii) change in stool consistency [18]. Additional symptoms that are common but not specific to IBS include excessive straining during defecation, urgency, bloating, abdominal distension, feelings of incomplete evacuation, and mucus bowel movements [18].

The comorbidity rate among patients suffering from IBS is relatively high. Patients who suffer from IBS complain of significantly reduced quality of life (QoL), which is comparable to other chronic diseases like diabetes mellitus and hepatitis [15]. Furthermore, IBS patients exhibit co-occurring mental health issues, i.e., anxiety and depression, more often compared to healthy individuals [19]. Moreover, these patients present other symptoms, which might be located in the gastrointestinal tract (i.e., dyspepsia) or other areas, such as fibromyalgia (FM), migraine headaches, fatigue, sleep problems, dizziness, irritability, lower urinary tract symptoms (such as painful bladder syndrome—PBS), and TMDs [1,6,13,20,21,22,23].

### 1.3. Correlation Between TMD and IBS

Both disorders have similar risk factors, like anxiety, which triggers visceral hypersensitivity implicated in the pathogenesis of both TMD and IBS. Furthermore, TMD and IBS patients often report additional (widespread) pain, which is not correlated with a primary condition. It ultimately results in overlapping pain syndromes, complicating diagnosis [4,24,25,26,27,28]. Importantly, studies show that the simultaneous therapeutic approach to these co-morbid disorders is more effective than treating them separately. However, it should be emphasized that the association between these conditions is still not sufficiently explored.

This scoping review aimed to investigate the association between temporomandibular joint disorder (TMD) and irritable bowel syndrome (IBS). This review explored the correlation between these conditions across various dimensions, including their pathology, prevalence, and severity, while also examining the potential similarities in how the nervous and endocrine systems impact them. This review specifically focuses on the multiple connections strictly between TMD and IBS, which has not been a subject of an original scoping (or any) review since 2020 [4].

## 2. Materials and Methods

A thorough search was conducted across PubMed, SCOPUS, Web of Science, EMBASE, and Google Scholar digital databases. Research solely in English and Polish, published after 2000, was considered. The search strategy utilized variations of “irritable bowel syndrome” and ‘temporomandibular disorders’. The Boolean operator ‘and’ was employed to merge the search terms of irritable bowel syndrome (IBS) and temporomandibular disorders (TMDs) such as “coexistence”, “TMD comorbidity”, “IBS comorbidity”, “risk of TMD”, “risk of IBS”, “TMD overlapping conditions”, “IBS overlapping conditions”, “TMD exacerbating factors”, and “IBS exacerbating factors”.

Initially, a total of 20,009 articles were identified. The articles underwent a screening process by two of the authors. Subsequently, the remaining articles underwent a full-text assessment to ascertain their eligibility for inclusion. Full texts were searched on the National Center for Biotechnology Information website. Each article underwent a thorough full-text assessment.

**Figure 1:**
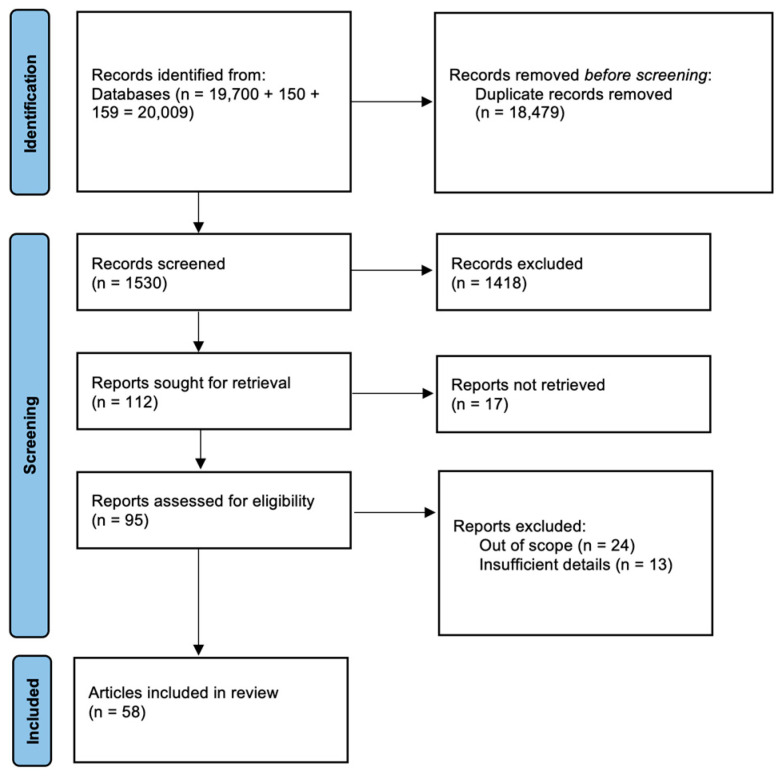
Search flow diagram based on PRISMA guidelines.

Following the selection criteria application, a total of 58 clinical papers met the eligibility requirements for inclusion in the review. The full list of papers along with their description is presented in the Supplementary Materials File S1. The PRISMA flow diagram presents the article assortment process (Figure 1). The inclusion and exclusion criteria for the articles are presented in Table 1.

## 3. Results

### 3.1. The Incidence of TMD in Patients with IBS

It was proven that each of these two conditions contributes to the development of the other. IBS patients presented more than three times greater risk of TMD compared to a healthy control group. Moreover, the probability of having TMD was comparable in every IBS subtype [1]. The frequency of TMD was three times higher in people with IBS than in people without IBS, but the result decreased on adjustment for demographic characteristics [29]. IBS manifestations were confirmed to be relevant predictors of TMD occurrence [29]. It turned out that patients who presented six or more (out of ten) IBS symptoms also presented a greater incidence rate of TMD than patients without any IBS symptoms [29].

### 3.2. The Incidence of IBS in Patients with TMD

When we take into consideration the opposite correlation, it turns out that the incidence of IBS among TMD patients is significantly greater than in individuals without TMD. The risk of IBS is more than six times greater in TMD cases [2]. This thesis is also confirmed by the fact that TMD patients, apart from complaining of more current back pain, a greater number of back pain episodes in the past year, and more functional disorder-related symptoms, also more often report Rome criteria for IBS [30]. The correlation between TMD and IBS was previously researched [1,6,21,22,23,30]. Hence, it has not been exhaustively described in the literature.

Interestingly, one study presented an animal model of chronic visceral hypersensitivity, which was a specific symptom of IBS [24]. In this experiment, ovariectomized rats with or without estradiol replacement were exposed to craniofacial muscle injury, followed by stress. In rats with estradiol administration followed by distal somatic injury and stress, visceral hypersensitivity lasting for months was observed. The stress paradigm alone provokes hypersensitivity for eight days, while muscle injury before stress exposure triggers visceral hypersensitivity that persists for weeks longer. Muscle injury followed by stress is a characteristic reported in TMD patients; thus, this study confirmed that TMD and IBS often occur as a comorbid condition [24].

### 3.3. The Influence of TMD on IBS Intensity

Research that focused on the influence of TMD on IBS intensity was inconsistent. In one of them, the substantial correlation between TMD and IBS severity was reported [2]. TMD patients have a greater risk of moderate and severe forms of IBS. Interestingly, there is no such dependence when it comes to mild IBS [2]. However, other research did not confirm that kind of relationship [1].

Patients who suffered from IBS accompanied by other functional disorders had more severe IBS symptoms than the ones who suffered from this illness only [20,31]. According to the analysis, facial pain that was present in TMD patients was positively related to abdominal pain [1]. However, other research obtained contradictory results [2]. After including Axis II variables, the only predictors of abdominal pain were nonspecific physical symptoms. It suggests that such patients suffer from a general, systemic disorder called central sensitivity syndrome (CSS) that involves different body regions and, for example, it is called “TMD” in the orofacial region and “IBS” in the intestinal region. It embraces the hypothesis that TMD and IBS are not separate entities but symptoms of one ailment [2].

### 3.4. The Influence of IBS on TMD Intensity

In a study performed by Dahan et al. [6], the number of comorbidities was associated with longer TMD pain duration and higher intensity. However, while migraine and chronic fatigue syndrome were shown to correlate with pain duration and intensity among TMD patients, no such correlation for IBS was found with these symptoms [6]. Nevertheless, in another study, IBS patients suffered from more TMJ crepitation (20.9 vs. 10.5, *p* = 0.10) and significantly more facial pain (37.4% vs. 19.3%, *p* = 0.02), TMJ locking (13.2% vs 3.5%, *p* = 0.05), and TMJ clicking (41.8% vs. 17.5%, *p* = 0.002) than did healthy individuals [1].

### 3.5. Pain

To consider possible reasons for the IBS and TMD association, it is desirable to indicate that many patients with any of these conditions report additional pain, which is perceived as not connected to the earlier complaint [32,33]. That results in comorbid or overlapping disease entities. Overall, in patients with chronic pain syndromes, the incidence of comorbidity totaled 50%. It renders marked pain management problems [21,24]. In TMD patients, apart from IBS, others were also frequently observed: migraine and chronic fatigue syndrome, fibromyalgia, vulvar vestibulitis syndrome, anxiety, and depressive disorders, as well as chronic pelvic pain [2,24,34]. Similarly, functional syndromes often co-occur in IBS patients, such as migraine, chronic fatigue syndrome, chronic pelvic pain, fibromyalgia, depressive syndromes, anxiety disorders, and TMD [13,17,20,31,35]. Moreover, patients who suffer from both IBS and TMD have greater pain sensitivity [1,4,36].

Many of these entities are included in a new category for a group of interdependent diseases called central sensitivity syndrome (CSS), whose etiology involves central sensitization (CS). CS is a term relating to a physiological phenomenon that involves improper, severe intensification of pain in the central nervous system with abnormalities in descending and ascending pathway activity [37].

People with functional pain syndromes had modified CNS processing, which was a contributing factor. It might indicate a mechanism for increased comorbidities [38,39]. Some articles revealed that people who suffered from TMD or IBS had deficiencies in pain modulation systems [36,40]. Patients suffering from TMD and IBS complained of increased sensitivity to heat pain and displayed impaired pain modulation systems like Diffuse Noxious Inhibitory Control (DNIC), whereas healthy individuals reported a marked reduction of pain due to DNIC [36,40]. It suggested that chronic pain patients may present dysfunction of endogenous pain inhibition systems. Moreover, patients suffering from TMD and IBS exhibited significantly higher anxiety levels than the healthy control group [36,41,42].

It was also proven that IBS patients presented a diminished ability to inhibit painful visceral stimulation and to involve DNIC systems. Similarly, in elderly people and in patients with fibromyalgia and chronic headaches, insufficient inhibition of somatic pain by the DNIC paradigm was noticed [22,36]. Generally, dysfunction of endogenous pain inhibitory systems was probably involved in increased sensitivity to experimental pain in TMD and IBS [36,43].

Neuronal hyperexcitability of dorsal horn neurons, which might result from impaired central modulation of the nociceptive activity, was regarded as one of the potential reasons that underlie the intensified pain processing in TMD and IBS patients [36]. As mentioned, central sensitization was a result of an extended hyperactivity of peripheral nociceptors and dorsal horn. It could result from neuroplastic changes in the nervous system. This was associated with a corresponding increase in wide dynamic neuron (WDR) activation in the spinal cord, which decreased while heterotopic conditioning stimulus via descending pathways occurred. Furthermore, pain levels during the sensitization paradigm (i.e., temporal summation) depended on C-fiber activity. Physiologically, the fibers were significantly prone to inhibition by DNIC [36]. Interestingly, it was proven that TMD and IBS patients with impaired nociceptive pain systems presented a common anatomic feature: the reduction in the gray matter within the limbic system and insula [4,44,45].

Surprisingly, decreased endogenous pain inhibition in patients enrolled in the study might result from extended expectations of pain [4]. Psychological factors, i.e., catastrophizing and the patient’s expectations, influenced pain modulation processes [36].

## 4. Discussion

### 4.1. The Influence of Stress and Anxiety

Psychological factors like stress and depression significantly influence the severity of pain syndromes [24,41,46,47,48]. Acute stress may display antinociceptive activity, for example, stress-induced analgesia [24], whereas visceral stimuli turned out to be pronociceptive. However, chronic stress is always considered pronociceptive. In patients with chronic pain syndromes, chronic stress may induce nociceptive episodes and increase pain complaints [7,22].

Patients with functional gastrointestinal disorders often also complain of affective disorders such as depression, anxiety, panic, and post-traumatic pain disorder [17,49]. Similarly, stress is reported as a significant factor in developing TMD, and it influences pain severity in TMD [50,51,52,53]. Therefore, since stress has an impact on many pain syndromes, it is understandable that individuals prone to one chronic condition display a higher risk of developing another condition in a stressful situation [24,54].

Anxiety is another significant factor associated with TMD; it is related to joint and muscle pain [55]. Children complaining of anxiety developed TMD 18 times more often [46]. Moreover, TMD patients exposed to environmental changes react more emotionally than healthy individuals. Indeed, among patients treated for this condition, approximately one-third report symptoms of depression [56]. Similar dependence occurs in IBS patients. Almost a third of IBS patients are affected by depression and anxiety disorders [57,58]. It indicates that there is a significant correlation between both TMD and IBS and psychiatric disorders, which should be taken into account during the course of treatment.

### 4.2. The Influence of Gender on TMD and IBS

TMD is approximately twice as prevalent in women than in men, and 80% of patients treated for TMD are women [59]. Interestingly, a higher D2:D4 digit ratio (a marker of higher estrogen compared to testosterone in utero) was associated with a greater incidence of first-onset TMD [29]. Therefore, gender proved to be a risk factor in chronic TMD since higher levels of estrogen predispose one to this disorder [1,10]. The acute form is equally common in both sexes [60]. TMD patients suffering from comorbidities were mostly women with myofascial TMD, a history of depression and/or anxiety, and experienced higher TMD pain intensity [6]. Conversely, recent research findings indicate that low estrogen levels may also exacerbate certain types of TMD [60]. For instance, TMJ degeneration is more prevalent in women over the age of 50. Interestingly, there is a decline in disc degeneration disorders within the same age group, while the incidence of this dysfunction peaks during childbearing age [60]. Approximately 60–70% of IBS patients are women, which may indicate that gender is also a risk factor for developing IBS [17]. Female patients most commonly develop IBS in the late teens to mid-forties, and the incidence of IBS decreases steadily with age, approaching the rate observed among men by the 7th decade of life. For comparison, the prevalence of IBS among men is fairly constant in the 20 to 70 years age range [17,61,62,63]. Women with IBS rate the severity and duration of abdominal pain or discomfort higher than their male counterparts. They are more likely to report constipation, bloating, and a feeling of incomplete evacuation, while for men, it is more typical to have diarrhea-associated symptoms. Women with IBS are also more likely to demonstrate symptoms of anxiety and depression than male IBS patients [62].

Generally, women are more sensitive to pain than men [62,64]. Estrogen can exert analgesic and hyperalgesic impact depending on the experimental conditions [62,65]. It can affect the CNS by modulating the production and action of neurotransmitters and influencing electrical excitability [62]. In animal research, stress and manipulation of sex hormones were reported to have an impact on nociceptive processing, particularly that of deep tissue. It resulted from its effect at various levels of the neuraxis [24]. Interestingly, in fertile women, increased levels of estrogen were associated with the elevated number of μ-opioid receptors in the brain regions related to pain processing [62,66]. There is also evidence that estrogen ameliorates recovery after chronic stress [6,62,67]. On the other hand, female IBS patients taking oral contraceptives (OCs), which contain estrogen and progestin, appear to have reduced levels of abdominal symptoms, especially dysmenorrhea [62,68,69]. However, gastrointestinal and non-gastrointestinal symptom patterns over the menstrual cycle are similar in IBS female patients, regardless of OC use or the predominant bowel pattern [61,62]. Animal studies proved that high ovarian hormone levels during pregnancy reduce somatic and visceral pain sensitivity [62]. Many chronic pain syndromes that are frequently associated with IBS are alleviated during pregnancy [52].

Decreasing or low levels of ovarian hormones in women (e.g., during menstruation) often lead to exacerbation of gastrointestinal (GI) symptoms [62,70]. The rectal pain threshold is also significantly lower in patients with IBS during menses compared to patients in other phases of the cycle. One-third of asymptomatic women experience GI symptoms at the time of menstruation. In the postmenopausal period, some of these symptoms, such as constipation and somatic discomfort, may be more prevalent [62]. Surprisingly, when it comes to the incidence of IBS, it turns out to be lower in women after menopause [62,71].

High levels of estrogen reduce TMD pain [60]. During pregnancy, the prevalence of TMD is 2–3 times lower than in nonpregnant age-matched women. Studies also demonstrate that orofacial pain is diminished during the third trimester and increases postpartum [60]. On the other hand, hormone replacement therapy (HRT) was reported to have no crucial effect on the prevalence of TMD [72]. The explanation for discordant results between pregnancy and HRT is that tissues may have altered sensitivity to fluctuating estrogen levels rather than to low or high concentrations [60]. Additionally, HRT in postmenopausal women is associated with an increased incidence of IBS. HRT may not only prolong IBS symptoms, but also trigger changes in gastrointestinal function in women not previously affected. Moreover, both current and past users indicate an increased risk of IBS, which is irrespective of treatment duration, regimen, or route of administration [62,73]. Interestingly, higher androgen levels associated with males probably protect against the development of chronic pain disorders. Moreover, testosterone demonstrates anti-inflammatory and analgesic properties. These factors indicate that differences in androgen levels may be the reason for gender inequalities when it comes to the risk of developing chronic pain diseases [62,69].

A huge influence on pain severity in TMD and IBS is psychological factors like stress and depression [24,50,74]. People with functional pain syndromes like TMD and IBS present modified central nervous system (CNS) processing and dysfunction, which might underlie the problem of huge comorbidity in these entities [24,39,40]. For both TMD and IBS, the female predisposition and the major role of estrogen are observed [62,65]. On the contrary, testosterone, with its anti-inflammatory and analgesic properties, is also a reason for gender inequalities when it comes to the risk of developing chronic pain diseases [62].

Nonspecific physical symptoms can be the only predictor of abdominal pain [1,2]. It can also be anticipated that rather than TMD patients being more prone to IBS, some patients suffer from a general, systemic disorder that manifests in different body regions as TMD and IBS [2]. These results highlight the significance of using Axis II questionnaires because clinical diagnosis is insufficient for understanding TMD patients. Psychosocial evaluation is also obligatory [2].

The rates of comorbidity in TMD and IBS patients are relatively high [1,24]. Furthermore, disorders that co-occur with both are similar [2]. That seems to confirm the hypothesis about existing CSS, a group of interrelated diseases with a common etiology related to CS [37]. Research suggests that treating co-morbid conditions like temporomandibular disorders (TMDs) and irritable bowel syndrome (IBS) together can be more effective than treating each disorder separately due to their common pathophysiological mechanisms [1,75]. Combining therapeutic approaches with psychological support can benefit synergistically [76,77].

A multidisciplinary approach involving dentists and gastroenterologists improves patient care by identifying and treating both disorders early. This collaborative model allows for comprehensive treatment plans that address the full scope of symptoms rather than focusing on isolated problems [34,78]. This approach enhances early diagnosis and treatment outcomes. Integrated care for TMDs and IBS is growing, but the optimal model remains unclear [34,79]. Future research should focus on effective interventions, such as shared medications, physical therapies, dietary changes, or psychological treatments. Large-scale, randomized controlled trials are needed to validate benefits and collaboration methods.

## 5. Conclusions

Psychological factors such as stress and depression significantly influence pain severity in functional pain syndromes like TMD and IBS. Both syndromes are proven to modify central nervous system processing, leading to high comorbidity. Both TMD and IBS are strongly correlated, with a potentially underestimated influence of visceral pain on TMD. However, it should be emphasized that the association between these conditions is still not sufficiently explored. Importantly, a simultaneous therapeutic approach to these co-morbid disorders is more effective than treating them separately. Cooperation between dentists and gastroenterologists, such as interviewing TMD patients for symptoms of IBS and vice versa, could significantly improve patient quality of life, but further research is needed for fully holistic diagnosis and treatment.

## Figures and Tables

**Table 1:** Inclusion and exclusion criteria.

Inclusion Criteria	Exclusion Criteria
Research on temporomandibular disorders (TMDs)	Articles in language other than English or Polish
Research on irritable bowel syndrome (IBS)	Research group smaller than 50 people
Research on associations between TMD and IBS	Publication before 2000
Research on TMD and IBS comorbidities	No full text available
Publication after 2000	Same data published at different time

## Supplementary Materials

The following supporting information can be downloaded at: https://www.mdpi.com/article/10.3390/jcm13237326/s1, Supplementary File S1: Papers included in the review list.
